# Tumefactive primary central nervous system vasculitis mimicking a brain metastasis in a patient with kidney cancer

**DOI:** 10.1055/s-0044-1786762

**Published:** 2024-05-13

**Authors:** Leonardo Furtado Freitas, Osorio Lopes Abath Neto, Joan E. Maley, Nitesh Shekhrajka, Bruno A. Policeni

**Affiliations:** 1 University of Iowa Hospitals and Clinics, Radiology Department, Neuroradiology Division, Iowa City Iowa, United States.; 2 University of Iowa Hospitals and Clinics, Pathology Department, Iowa City Iowa, United States.

**Figure 1:**
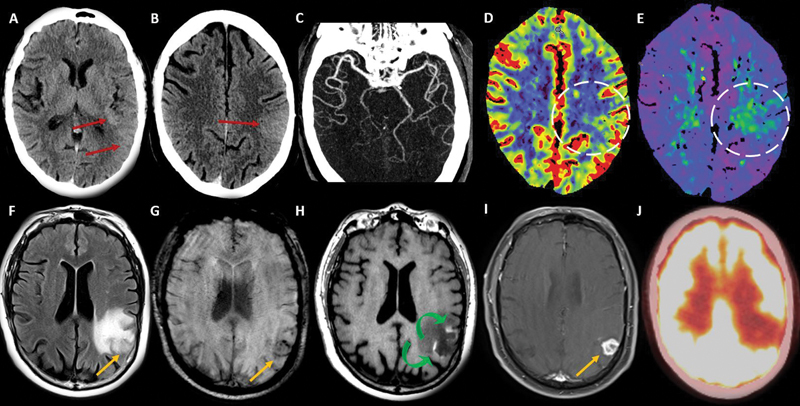
Head computed tomography (CT) stroke protocol ( **A-E** ) at the initial presentation. Subtle left parietotemporal and posterior insular hyperdense and swollen cortex ( **red arrows** ), with local sulci effacement. The head CT demonstrated normal left middle cerebral artery (MCA) opacification ( **C** ) and the perfusion maps showed mild increased cerebral blood flow (CBF) ( **D** ) and (time to drain) TTD ( **E** ). At the one-month follow-up a brain magnetic resonance imaging (MRI) scan ( **F-I** ) demonstrated a cortical/subcortical round lesion ( **orange arrows** ) with peripheral microbleeds ( **G** ) and irregular enhancement ( **I** ), surrounded by vasogenic edema and laminar cortical necrosis ( **curved green arrows in H** ). There was no high fluorodeoxyglucose (FDG) uptake in this area on the positron-emission tomography–computed tomography (PET-CT) scan ( **J** ).

**Figure 2:**
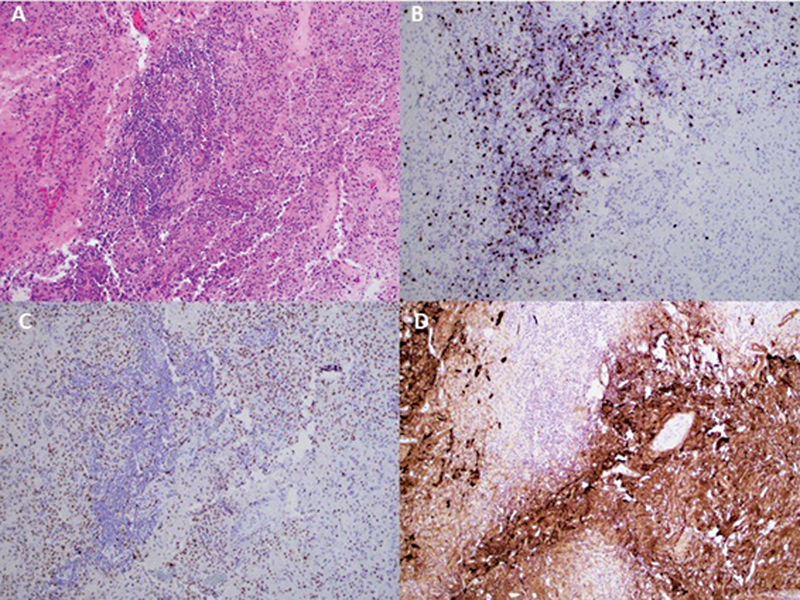
Brain parenchyma containing a dense inflammatory infiltrate with a predominant perivascular distribution ( **A** ). The infiltrate was composed of an admixture of numerous CD8-positive T-cells ( **B** ), scattered CD20-positive B-cells ( **not shown** ), and diffusely distributed histiocytes highlighted by PU.1 ( **C** ). Glial fibrillary acidic protein (GFAP) ( **D** ) demonstrated extensive and marked reactive astrogliosis around the inflammatory infiltrates.

A 71-year-old male patient with papillary renal cell carcinoma (RCC) presented with confusion, right hemiparesis, and aphasia. A Head computed tomography (CT) scan ( Figure 1 ) demonstrated a hyperdense swollen cortex on the left side, patent left middle cerebral artery (MCA), and abnormal perfusion. A brain magnetic resonance imaging (MRI) scan and a positron-emission tomography–computed tomography (PET-CT) scan ( Figure 1 ) at one month of follow-up showed a round lesion with low fluorodeoxyglucose (FDG) uptake with enhancement and laminar cortical necrosis. The differentials included brain metastasis. After the surgery, the pathology ( Figure 2 ) favored vasculitis. This pseudotumoral presentation of central nervous system (CNS) vasculitis is always challenging, 1 especially in cancer patients. 2 The analysis enabled us to consider that it was an acute/subacute and progressive vascular injury, with an evolving enhancing necrotic lesion due to the blood-brain barrier disruption and laminar cortical necrosis.
